# Carbonic Anhydrase Inhibition and the Management of Hypoxic Tumors

**DOI:** 10.3390/metabo7030048

**Published:** 2017-09-16

**Authors:** Claudiu T. Supuran

**Affiliations:** Università degli Studi di Firenze, Dipartimento Neurofarba, Sezione di Scienze Farmaceutiche e Nutraceutiche, Via U. Schiff 6, 50019 Sesto Fiorentino, Florence, Italy; claudiu.supuran@unifi.it; Tel.: +39-055-457-3729; Fax: +39-055-457-3385

**Keywords:** tumor, metabolism, carbonic anhydrase, isoforms IX and XII, inhibitor, sulfonamide, antibody

## Abstract

Hypoxia and acidosis are salient features of many tumors, leading to a completely different metabolism compared to normal cells. Two of the simplest metabolic products, protons and bicarbonate, are generated by the catalytic activity of the metalloenzyme carbonic anhydrase (CA, EC 4.2.1.1), with at least two of its isoforms, CA IX and XII, mainly present in hypoxic tumors. Inhibition of tumor-associated CAs leads to an impaired growth of the primary tumors, metastases and reduces the population of cancer stem cells, leading thus to a complex and beneficial anticancer action for this class of enzyme inhibitors. In this review, I will present the state of the art on the development of CA inhibitors (CAIs) targeting the tumor-associated CA isoforms, which may have applications for the treatment and imaging of cancers expressing them. Small molecule inhibitors, one of which (SLC-0111) completed Phase I clinical trials, and antibodies (girentuximab, discontinued in Phase III clinical trials) will be discussed, together with the various approaches used to design anticancer agents with a new mechanism of action based on interference with these crucial metabolites, protons and bicarbonate.

## 1. Introduction

A salient feature of many tumors is the fact that they are hypoxic and acidic compared to normal tissues of the same type. This has been known for many decades as the Warburg effect [1,2] but has been explained at the molecular level only recently, after the discovery of a transcription factor regulating these phenomena, the hypoxia inducible factor 1α, HIF-1α [3,4,5].

**Figure 1:**
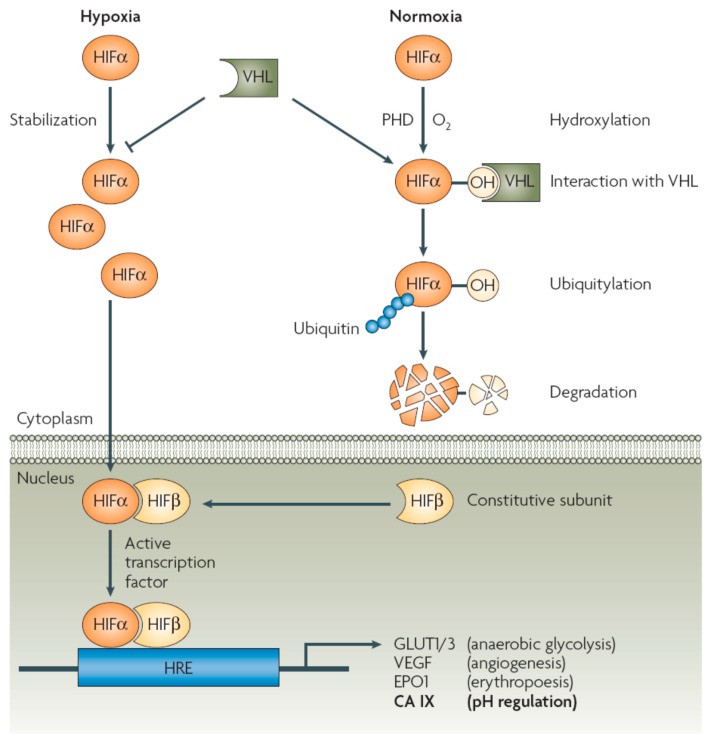
Mechanism by which the transcription factor HIF-1α (abbreviated as HIFα) orchestrates the overexpression of proteins involved in aerobic glycolysis, angiogenesis, erythropoesis and pH regulation in hypoxic tumors. In normoxia HIFα is hydroxylated at a Pro residue and targeted for degradation by the proteasome (PHD, prolyl-hydroxylase; VHL, von Hippel-Lindau factor, HRE, hypoxia responsive element). In hypoxia, its accumulation leads to overexpression of the proteins involved in tumorigenesis mentioned above [5,6,7,8].

As seen from Figure 1, in normoxic conditions HIF-1α is unstable, being degraded rapidly by a well understood biochemical process: under the action of prolyl hydroxylases (PHD), a proline residue from the transcription factor is hydroxylated, being then recognized by a protein possessing ubiquitin ligase E3 activity, more precisely the von Hippel Lindau protein (VHL), which targets it to ubiquitylation and degradation within the proteosomes (Figure 1) [5,6,7,8].

However, in hypoxia, which as mentioned above is frequent in many tumor cells [1,2,3], an accumulation of HIF-1α occurs, followed by its translocation from the cytosol to the nucleus, where it forms a dimer with a constitutive subunit, HIF-1β, leading to an active transcription factor, which, by interaction with a hypoxia responsive element (HRE) found on different genes, leads to overexpression of proteins involved in aerobic glycolysis (such as, for example, the glucose transporters GLUT1-3), angiogenesis (such as, for example, the vascular endothelial growth factor, VEGF), erythropoesis (such as, for example, erythropoetin 1) and pH regulation (such as the tumor-associated enzymes CA IX and XII) [5,6,7,8,9,10,11].

**Figure 2:**
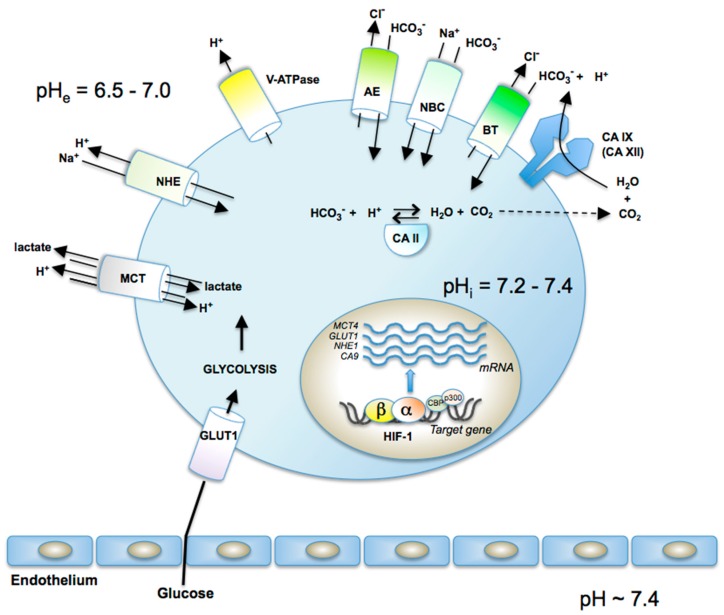
Proteins involved in pH regulation in tumors: GLUT1, the glucose transporter isoform 1; MCT, monocarboxylate transporter, which extrude lactic acid and other monocarboxylates formed by the glycolytic degradation of glucose; NHE, sodium-proton exchanger (Na^+^–H^+^ antiporter); V-ATPase, plasma membrane proton pump; AE, anion exchanger (chloride-bicarbonate exchanger); NBC, sodium bicarbonate channels; BT, bicarbonate transporter; CA II (cytosolic) and CA IX/XII, which catalyze CO_2_ hydration to bicarbonate and protons [4,5,6,7,8,9,10,11,12,13,14,15].

The overexpression of these proteins has profound effects on the metabolism of cancer cells, which on one hand are deprived of oxygen for the normal metabolism involving the oxidative phosphorylation [1,2], and on the other one, have an enhanced uptake of glucose (due to the overexpression of the glucose transporters GLUT1-GLUT3, which import the sugar within the cell), which cannot undergo the oxidative pathways for the generation of ATP [5,6,7,8]. Thus, an alternative pathway, the glycolytic one, occurs, with the formation of pyruvic (and lactic acids) from glucose, which generates less ATP (compared to the oxidative pathway), but which seems to be enough for the cancer cells to survive in hypoxic conditions [1,2,3,4]. The formed organic acids are extruded from the cells through the monocarboxylate transporters MCT1-MCT4 (some of which are overexpressed in tumors [4]), leading to an acidification of the extracellular milieu, up to pH values as low as 6.5 [4,5,6,7,8]. Additional perturbations of the extra- and intracellular pH equilibrium of the tumor cells are also furnished by other proteins which are involved in this process (Figure 2), among which the sodium-proton exchanger (Na^+^–H^+^ antiporter) NHE, which may import or export protons in exchange for sodium ions, the plasma membrane proton pump H^+^-ATPase (V-ATPase), the various isoforms of the anion exchangers (chloride-bicarbonate exchangers) AE1–AE3, the sodium bicarbonate channels NBCs, which transport sodium and bicarbonate out of the cell or import it within the cell, various other bicarbonate transporters BT, as well as several isoforms of the metalloenzyme CA, such as the cytosolic CA II, and the transmembrane CA IX/XII, which efficiently catalyze CO_2_ hydration to bicarbonate and protons [4,5,6,7,8,9,10,11]. By the coupling of all these effects, a slightly alkaline intracellular pH is achieved (of around 7.2) and an acidic extracellular pH of the tumor is formed, with values as low as 6.5 [4,5,6,7,8,9,10,11] (Figure 2). The extracellular acidosis (coupled with the hypoxia) is beneficial for the growth of the tumor cell and impairs the growth of the normal cells, leading thus to a massive proliferation, invasion and subsequently metastasis of the primary tumors [12,13,14,15].

Data of Figure 1 and Figure 2 show the multitude of proteins involved in these processes, which in the end lead to features of the tumor cells which are quite different from those of the normal ones, and could thus be exploited for designing novel anticancer therapies. Among those who proposed this approach for the first time was Pouysségur et al. [4] who initially considered the NHE inhibitors as the most interesting pharmacological agents for interfering with tumor hypoxia/acidosis [16]. However, the significant toxicity of this class of drugs, or the lack of isoform-selective ones for other proteins involved in these processes (such as the MCTs, AEs, V-ATPase, etc. [17,18]) led to most of the work being concentrated on the metalloenzyme involved in pH regulation, i.e., the carbonic anhydrase (CA, EC 4.2.1.1) [7,8,19,20]. It should be however mentioned that H^+^/K^+^-inhibitors of the omeprazole type were shown to possess, alone or in combination with CA inhibitors (CAIs) significant antitumor effects [21,22,23]. Here I shall review the field of the CAIs as theragnostic agents for the management of hypoxic, metastatic tumors, without considering the other valuable approaches found in the literature which target other of the many proteins involved in these processes, and which have been reviewed by other researchers [6,13,16].

## 2. Validation of CA IX/XII as Antitumor Drug Targets

**Figure 3:**
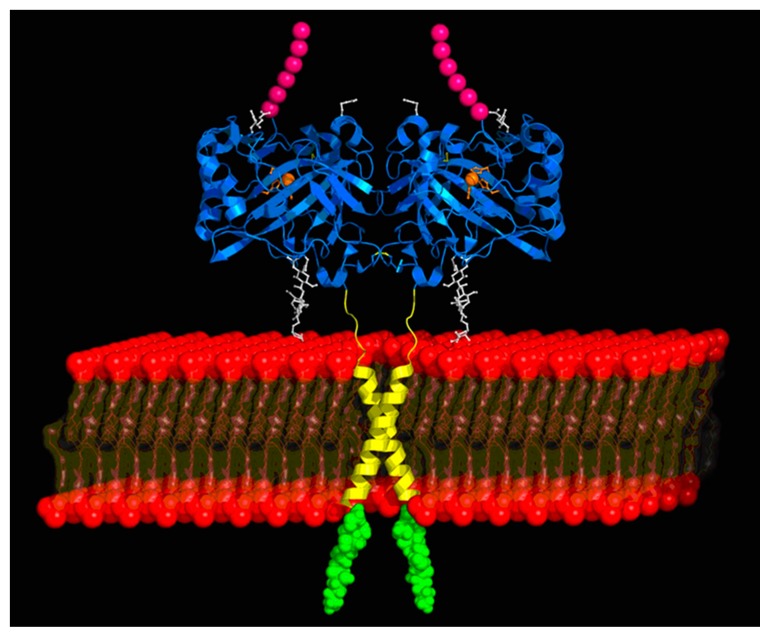
CA IX X-ray crystal structure of the catalytic domain (in blue), the PG domain (cartoon in pink), plasma membrane (in red), the transmembrane domain in yellow (modeled) and the intracytosolic tail (modeled, in green) [29].

CA IX was discovered by Pastorek et al. in 1994 [10] and CA XII by Tureci et al. in 1998 [11], and it became immediately obvious that they differ considerably from other members of this family of proteins, which includes 15 isoforms in humans, hCA I-hCA VA, hCA VB, hCA VI-hCA XIV [7,8,20]. The first unusual feature of CA IX and XII was that the two enzymes are transmembrane, multi-domain proteins incorporating a short intra-cytosolic tail, a transmembrane short domain, and an extracellular catalytic domain, rather homologous to the one found in the cytosolic, mitochondrial, secreted or membrane-anchored CA isoforms known at that time [10,11,24,25,26,27]. Furthermore, CA IX has an additional domain at its *N*-terminus, termed the proteoglycan (PG) domain, which seems to play important functions connected with the role of CA IX in tumorigenesis being present only in this CA isoform [28,29] (Figure 3). In fact, all domains of this molecule, the intracellular tail [30], the catalytic domain [25,29] and the PG domain play diverse functions in tumorigenesis, making CA IX one of the key proteins involved in such processes in hypoxic tumors [7,8,15,17,24,25,26,27,28,29,30]. It is also interesting to note that CA IX seems to be an even more complicated protein: recent proteomic/interactomic studies suggests that at a stage in the cell’s life CA IX possibly has a nuclear localization [31], interacting with proteins involved in nuclear/cytoplasmic transport processes, gene transcription, and protein stability, among which cullin-associated NEDD8-dissociated protein 1 (CAND1), which is itself involved in gene transcription and assembly of ubiquitin ligase complexes [32]. The precise role of these interactions of CA IX with this type of proteins is poorly understood at this moment but may lead to significant drug design developments in the future.

Returning to the main function of CA IX/XII, that of catalyzing the hydration of CO_2_ to bicarbonate and protons [7,8], the validation of these proteins as drug targets followed the usual steps that most drug targets experience. They are summarized below:

(1) recombinant CA IX and XII were shown to possess a significant catalytic activity (in vitro) for the physiologic reaction (hydration of carbon dioxide to bicarbonate and protons), being among the most effective catalysts known in nature, with the following kinetic parameters: for human (h) CA IX (full length): k_cat_ of 1.1 × 10^6^ s^−1^, k_cat_/K_M_ of 1.5 × 10^8^ M^−1^ s^−1^ [24], whereas for hCA XII (catalytic domain) these parameters are k_cat_ of 4.2 × 10^5^ s^−1^, k_cat_/K_M_ of 3.5 × 10^7^ M^−1^ s^−1^ [33].

(2) potent in vitro CAIs of the sulfonamide type have been identified for both hCA IX [34] and hCA XII [33], followed by a large number of drug design studies of such agents [35], which have been reviewed recently and will be not detailed here [36,37,38]. As a consequence of such studies a large number of sulfonamide, sulfamate and sulfamides showing effective hCA IX/XII inhibitory potency (in vitro) and sometimes also some selectivity for inhibiting these two isoforms over the cytosolic, off-target and widespread ones hCA I and II, became available for in vivo studies [33,34,35,36,37,38,39]. 

The drug design of CAIs targeting isoform IX were highly favored by the report of the X-ray crystal structure of the protein (its catalytic domain) by De Simone’s group in 2009 [25]. This 3D structure allowed the identification of similarities and differences between CA IX and the other members of the family, which led to the identification soon thereafter of highly isoform-selective inhibitors belonging to a variety of chemical classes, such as the sulfonamides, sulfamates, sulfamides, coumarins, polyeamines, etc. [36,37,38].

(3) Pastorekova’s group [29] demonstrated the role of CA IX in extracellular acidification of hypoxic tumors, and the possibility to reverse this effect by inhibiting the enzyme activity with sulfonamides. Furthermore, in the same studies it was observed that a fluorescent potent sulfonamide CA IX/II inhibitor accumulated only in the hypoxic cells, whereas it did not bind in cells expressing CA IX, but in normoxic conditions [29,39]. This effect has been explained as being due to the PG domain of the protein, which in normoxic conditions closes the active site. The opening of the active site is triggered by hypoxia, making it available for inhibitors to bind, but only in hypoxic conditions [39]. This makes CA IX an ideal drug target, as this phenomenon will lead to the inhibition of only the CA IX present in tumors, leading thus to drugs with fewer side effects compared to the classical chemotherapeutic agents [29,39].

(4) Dubois et al. [40,41] then published the proof-of-concept studies showing that in xenograft animal models of hypoxic tumors it is possible to image the hypoxic regions rich in CA IX/XII by using fluorescent sulfonamide CAIs possessing the same structural elements as the compounds used in the study of Pastorekova’s group, mentioned above [29].

(5) The first study showing an in vivo antitumor effect due to CA IX inhibition was from Neri’s group [42], followed shortly thereafter by similar studies from different laboratories, on diverse models and cancer types, which demonstrated that sulfonamide/sulfamate [42,43,44,45,46] or coumarin [47] CA IX/XII inhibitors have a profound effect in inhibiting the growth of the primary tumors and the metastases expressing CA IX/XII. Probably the most interesting studies are those from Dedhar’s group [44,45,47] who rigorously showed the involvement of CA IX/XII in the antitumor/antimetastatic effects of the inhibitors of the sulfonamide or coumarin types. In fact, as it will be shown shortly, one of the compounds described in such studies progressed to clinical trials and completed Phase I trials in 2016 [45].

(6) Dedhar’s group [48] also discovered another important phenomenon connected to CA IX/XII inhibition, i.e., the depletion of cancer stem cell population within the hypoxic tumors, which is considered to be a very positive feature of an antitumor agent, considering the fact that most such therapies lead to an increase of this stem cell population, hypothesized to be one of the reasons for the recurrence of some cancers [49]. The same group recently elucidated [50] the mechanism used by the hypoxic tumors for invasion, which reinforces the role played by CA IX in tumor progression and clinical outcome of cancer patients harboring CA IX-positive tumors. This relevant study demonstrated an association between CA IX and matrix metalloproteinase 14 (MMP14), with the first protein furnishing H^+^ ions used in the proteolytic cleavage of collagen mediated by MMP14, which leads to tissue degradation. This study showed that CA IX is one of the metabolic components of the cellular migration and invasion mechanisms in hypoxic tumors, and provides new mechanistic insights into the role played by this enzyme in tumor cell biology, with the possibility to design dual agents, targeting both these enzymes (CA IX and MMP14) as new antitumor drugs [50].

## 3. Small Molecule CA IX/XII Inhibitors as Antitumor Agents

Among the huge number of sulfonamide, sulfamate, sulfamide and coumarin CA IX/XII inhibitors reported to date [4,7,34,35,36,37,38], few compounds were investigated in detail in animal tumor models, and only one such derivatives, SLC-0111 (also known as WBI-5111) progressed to clinical trials [45,51].

**Figure 4:**
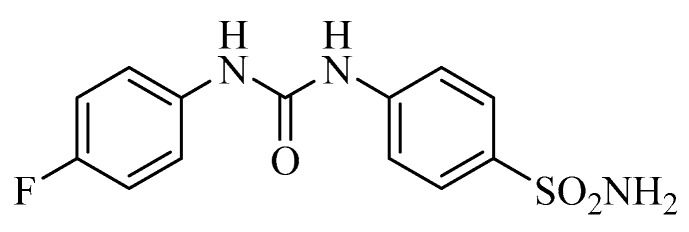
Structure of SLC-0111 (WBI-5111), the sulfonamide CA IX/XII inhibitor in Phase I/II clinical trials.

As seen from Figure 4, SLC-0111 is a simple, ureido-substituted benzenesulfonamide derivative which has significant hCA IX and XII inhibitory properties in vitro (K_I_s of 45 nM against hCA IX and of 4.5 nM against hCA XII), being much less effective as an inhibitor of hCA I and II, widespread cytosolic CAs in many organs [45]. The CA IX/XII-selective inhibitory properties of this sulfonamide and of some of its congeners were explained at the molecular level by using X-ray crystallography of enzyme-inhibitor adducts [52]. This study allowed to observe that the tail of the inhibitors (in the case of SLC-0111, the tail is a 4-fluorophenyl moiety) adopts very different conformations when the sulfonamide is bound within the enzyme active site cavity, and is orientated towards the exit of the cavity, which is the most variable part of the different CA isoforms present in mammals [52]. As a consequence, this class of sulfonamide CAIs show some of the highest selectivity ratios for inhibiting the tumor-associated over the cytosolic isoforms [52]. In vivo studies showed SLC-0111 to potently inhibit the growth of tumors harboring CA IX/XII, whereas tumors that did not express these enzymes were unaffected [44,45]. The metastases formation was also inhibited in the T4 murine breast cancer model [44], and important antitumor effects were observed also in combination with other anticancer agents used clinically, such as paclitaxel, doxorubicine, etc. [44,45]. As mentioned above, a notable depletion of the cancer stem cell population was also evident after the treatment with this compound. Although the results of the Phase I clinical trial are not yet published, the compound has been scheduled for Phase II trials which will start late in 2016 [51].

Although there are many other highly effective in vitro CA IX inhibitors reported so far, only a few of them were investigated in vivo in details. In one such study [53], important inhibition of growth of osteosarcoma was observed after inhibiting CA IX with positively charged pyridinium sulfonamides, suggesting their potential use for this refractory, difficult to treat tumor. In another study, [54], the CA IX and AP endonuclease-1/redox effector factor 1 (APE1/Ref-1) dual targeting was shown to be synergistic in pancreatic ductal adenocarcinomas (PDACs), another difficultly treatable tumor. A different and innovative approach has been used on the other hand by Neri’s group [55], who conjugated maytansinoid DM1, a cytotoxic natural product payload, to a sulfonamide, more precisely a derivative of acetazolamide (a clinically used CAI drug for decades [7,8]), as targeting ligand for CA IX recognition. This conjugate molecule exhibited a potent in vivo antitumor effect in SKRC52 renal cell carcinomas [55].

It is probable that many other small molecule CA IX/XII inhibitors may enter soon in clinical trials, but probably, most researchers/companies wait for results of the clinical trials of the first-in-the-class such compound (SLC-0111) to be released.

## 4. Antibodies Targeting CA IX and XII as Antitumor Agents

### 4.1. Anti-CA IX Antibodies

The renal cell carcinoma (RCC)-associated protein G250 was recognized by its discoverers to be an anti-CA IX monoclonal antibody (Mab) and proposed as a possible antitumor target for RCC [56]. Indeed, G250, formulated as chimeric IgG1 monoclonal antibody and denominated girentuximab, was the first CA IX inhibitor to enter clinical trials [57], being actually in Phase III, although it seems that its development has been interrupted due to lack of efficacy [58]. Thus, no other details will be discussed about this Mab, but Pastorekova’s group [59,60] proposed several interesting approaches based both on antibodies that inhibit the catalytic activity as well as those that target the PG domain of CA IX (and do not inhibit the CO_2_ hydrase activity of the enzyme). For example the mouse monoclonal antibody VII/20 was shown to bind to the catalytic domain of CA IX, leading to an efficient receptor-mediated internalization of the antibody-enzyme conjugate, which is the main process that regulates abundance and signaling of cell surface proteins [60]. This internalization has a considerable impact on immunotherapy and in this particular case elicited significant anticancer effects in a mouse xenograft model of colorectal cancer [60]. The same group [59] demonstrated that the monoclonal antibody M75 (targeting the PG domain of CA IX and widely used as a reagent in immune-histochemical studies [10,17]) can be encapsulated into alginate microbeads or microcapsules made of sodium alginate, cellulose sulfate, and poly(methylene-co-guanidine), which afforded a rapid M75 antibody release at pH 6.8 (characteristic of the acidic tumors) compared to pH 7.4 (the physiologic, normal pH) [59]. 

### 4.2. Anti-CA XII Antibodies

There are far fewer studies to target CA XII with Mabs compared to CA IX. The most significant one comes from Zeidler’s group [61] who discovered 6A10, the first monoclonal antibody that binds to the catalytic domain of CA XII and also acts as an inhibitor of the enzyme. 6A10 was shown to be a low nanomolar CA XII inhibitor and to inhibit the growth of tumor cells in spheroids and in vivo, in a mouse xenograft model of human cancer [61,62].

## 5. Imaging CA IX/XII Positive Tumors

The initial imaging strategy (after the fluorescent sulfonamides used for the proof-of-concept study mentioned above, which cannot be used to image human cancers [41]) was to incorporate ^99m^Tc or ^18^F as positron-emitting isotopes in the molecules of sulfonamide or coumarin CAIs in order to obtain agents useful for positron emission tomography (PET) [63,64,65,66]. The initial sulfonamides or coumarins labeled with these isotopes were not highly efficient imaging agents, probably due to pharmacokinetic-related problems. For example the SLC-0111 analog labeled with ^18^F as well as a coumarin derivative incorporating the same isotope, although highly potent as in vitro CA IX inhibitors in vivo, in HT-29 (colorectal) xenografts in mice did not accumulate in the tumor, but were principally present in the blood, liver and nose of the animals, making them inappropriate as PET agents. However, the next generation inhibitors labeled with ^18^F (trimeric sulfonamides [67] or positively-charged sulfonamides [68]) or ^68^Ga-labelled sulfonamides (originally reported by Bénard’s group [69] and soon thereafter by Poulsen’s group [70]) showed that such ^68^Ga-polyaminocarboxylate chelator-conjugated sulfonamides do accumulate preferentially within the hypoxic tumor, making them excellent candidates for clinical studies [69]. In HT-29 colorectal xenograft tumors in mice, the gallium-containing sulfonamides showed an excellent and specific tumor accumulation, coupled with a low uptake in blood and clearing intact into the urine, making them of great interest for further development [69,70].

Antibodies were also proposed as imaging agents for CA IX-positive tumors, originally by Neri’s group [71]. By using the phage technology, high-affinity Mabs targeting hCA IX were generated (denominated A3 and CC7) which were used for imaging purposes in animal models of colorectal cancer (LS174T cell line). Such imaging studies with the two anti-hCA IX Mabs disclosed by this group closely matched the pimonidazole (an azole agent which accumulates in hypoxic regions of tumors) staining of these tumors, furnishing the proof-of-concept study that, in addition to the small molecule CA IX inhibitors, the antibodies can also be used for non-invasive imaging of hypoxic tumors. There are in fact many other similar imaging studies of the Mab in clinical trials mentioned above, girentuximab, which has been labeled with various isotopes for these purposes. For example, ^111^In- [72], ^99m^Tc- [73] and ^124^I [74]—labeled girentuximab as well as dual-labeled Mab with a radionuclide and a fluorescence tag [75] have been developed and used for hypoxic tumor imaging with various degrees of success. However, antibodies have some problematic pharmacological aspects that must be considered attentively when used, and most probably small molecule CA IX/XII inhibitors may be more useful and easier to develop for a possible theragnostic agent targeting these enzymes.

## 6. Conclusions

Discovered at the beginning of the 90s, CA IX (and subsequently CA XII) were shown to possess crucial roles in tumorigenesis due to their involvement in the metabolism of hypoxic, acidic tumors. Overexpressed in tumor cells as a consequence of the HIF-1 cascade, these enzymes generate H^+^ and bicarbonate ions, the simplest metabolites known, from CO_2_ as substrate, being involved in many processes connected to tumorigenesis, from the regulation of the internal/external tumor cell pH, to migration, invasion, metastases formation as well as regulation of the cancer stem cell population. Many of these fascinating phenomena, discovered in the last decade, were shown to be useful for obtaining antitumor therapies/tumor imaging agents with a novel mechanism of action, by targeting these enzymes either with small molecule inhibitors or antibodies. The initial success of the clinical trials started with these agents, which is a continuing story, constitutes an excellent example of how fundamental research discoveries, thought to only explain some intricate biochemical/physiologic processes, may lead to innovative therapeutic strategies for fighting tumors.
